# Bacterial Antibiotic Resistance: The Most Critical Pathogens

**DOI:** 10.3390/pathogens12010116

**Published:** 2023-01-10

**Authors:** Carmelo Biondo

**Affiliations:** Department of Human Pathology, University of Messina, 98125 Messina, Italy; cbiondo@unime.it; Tel.: +39-0902213312

Antibiotics primarily act on bacterial growth by eliminating bacteria or preventing them from reproducing and spreading [1,2]. However, because they do so at different rates, the remaining bacteria may mutate if the dose is insufficient and resist antibiotic treatment through natural selection [2]. Antimicrobial resistance (AMR) is currently a serious threat to human health because antimicrobial drugs are becoming progressively less effective, and there are currently few new classes of antibiotics in the pharmaceutical chain [3]. The most recent new class of antibiotics was discovered in 1987; since then, only five new classes of antibiotics have been successfully introduced to the market [2]. Moreover, none of them target Gram-negative bacteria, which cause some of the most deadly and difficult-to-treat infections in hospital settings [4]. These and other important aspects related to AMR are discussed in the article “Bacterial Antibiotic Resistance: The Most Critical Pathogens”, published in *Pathogens*, volume 10, on 12 October 2021 by Mancuso G. et al. [2]. This article was a review that focused on the evaluation/discussion of individual articles concerning important aspects related to AMR. The review explores why and how antimicrobial resistance is becoming such a serious problem. The authors stress the importance of conducting accurate analyses and estimates of the impact of antimicrobial resistance on death, length of hospital stay, and healthcare costs to establish effective surveillance systems. In this regard, data obtained using predictive statistical models estimate that, if no action is taken, antimicrobial resistance could cause up to 10 million deaths, increasing global health care costs to $1 trillion per year by 2050 [3]. More than one million deaths per year are directly attributable to the six major pathogens for resistance-associated deaths (*Escherichia coli; Staphylococcus aureus; Klebsiella pneumoniae; Streptococcus pneumoniae; Acinetobacter baumannii* and *Pseudomonas aeruginosa*) [2]. 

The main objective of this review was to provide a detailed summary of the current status of AMR in these critical-priority bacteria. To make the complexity of the antibiotic resistance phenomenon as clear as possible, the review detailed both the mode of action and the mechanisms of resistance affecting commonly used antimicrobials. Finally, the review discussed the importance of implementing stewardship programs in light of the growing evidence that antimicrobial resistance is increasing, along with the increasing number of elderly patients with immunodeficiencies. We can stop the problem of antibiotic resistance only through immediate action, in order to avoid returning to a point when infectious diseases are the leading cause of death.
